# Assessment of the Spatial Distribution and Risk Associated with Fruit Rot Disease in *Areca catechu* L.

**DOI:** 10.3390/jof7100797

**Published:** 2021-09-24

**Authors:** Patil Balanagouda, Shankarappa Sridhara, Sandip Shil, Vinayaka Hegde, Manjunatha K. Naik, Hanumappa Narayanaswamy, Siva K. Balasundram

**Affiliations:** 1 Department of Plant Pathology, University of Agricultural and Horticultural Sciences, Shivamogga, Karnataka 577255, India; balupat007@gmail.com (P.B.); manjunaik2000@yahoo.com (M.K.N.); swamyareca@mail.com (H.N.); 2 Division of Crop Protection, ICAR-Central Plantation Crops Research Institute, Kasaragod, Kerala 671124, India; hegdev64@gmail.com; 3 Center for Climate Resilient Agriculture, University of Agricultural and Horticultural Sciences, Shivamogga, Karnataka 577255, India; 4 Research Centre, Division of Social Sciences, ICAR-Central Plantation Crops Research Institute, Mohitnagar, Jalpaiguri, West Bengal 735102, India; Sandip.iasri@gmail.com; 5 Department of Agriculture Technology, Faculty of Agriculture, Universiti Putra Malaysia, Serdang 43400, Malaysia

**Keywords:** fruit rot disease, point pattern analysis, surface interpolation, IDW, arecanut, disease risk estimation, spatial statistics

## Abstract

*Phytophthora meadii* (McRae) is a hemibiotrophic oomycete fungus that infects tender nuts, growing buds, and crown regions, resulting in fruit, bud, and crown rot diseases in arecanut (*Areca catechu* L.), respectively. Among them, fruit rot disease (FRD) causes serious economic losses that are borne by the growers, making it the greatest yield-limiting factor in arecanut crops. FRD has been known to occur in traditional growing areas since 1910, particularly in Malnad and coastal tracts of Karnataka. Systemic surveys were conducted on the disease several decades ago. The design of appropriate management approaches to curtail the impacts of the disease requires information on the spatial distribution of the risks posed by the disease. In this study, we used exploratory survey data to determine areas that are most at risk. Point pattern (spatial autocorrelation and Ripley’s K function) analyses confirmed the existence of moderate clustering across sampling points and optimized hotspots of FRD were determined. Geospatial techniques such as inverse distance weighting (IDW), ordinary kriging (OK), and indicator kriging (IK) were performed to predict the percent severity rates at unsampled sites. IDW and OK generated identical maps, whereby the FRD severity rates were higher in areas adjacent to the Western Ghats and the seashore. Additionally, IK was used to identify both disease-prone and disease-free areas in Karnataka. After fitting the semivariograms with different models, the exponential model showed the best fit with the semivariogram. Using this model information, OK and IK maps were generated. The identified FRD risk areas in our study, which showed higher disease probability rates (>20%) exceeding the threshold level, need to be monitored with the utmost care to contain and reduce the further spread of the disease in Karnataka.

## 1. Introduction

Arecanut or betel palm (*Areca catechu* L.) is an important plantation crop around the world that is widely cultivated in tropical regions of Asia [1,2,3]. In India, arecanut is largely cultivated in the plains and foothills of the Western Ghats and northeastern regions [4]. Data indicate that Karnataka, Kerala, Assam, West Bengal, Meghalaya, Nagaland, and Mizoram regions account for more than 90% of the total production in India [5,6]. Although the production of arecanut is confined to a few states, commercial products are extensively exported across the globe, and approximately 700 million people are dependent on arecanut worldwide [7,8]. Being a perennial crop, arecanut is highly vulnerable to numerous pathogens, including fungi [9,10,11], bacteria [12], phytoplasma [13,14,15], and viruses [16,17,18].

Arecanut production in India is threatened by fruit rot disease (FRD) caused by *Phytophthora meadii* (McRae), which is of greater concern in southern and eastern parts of the country [19,20,21]. In the Indian context, crop loss due to FRD represents about 10–90% of losses, with several reports estimating economic losses of up to 75% or complete destruction of palms [22]. In other reports, losses of 15–20% [23,24,25], 50–90% [26], and 72–350 kg nuts/acre [19] have been documented. Furthermore, [27] estimated yield losses to the tune of 10–50% in disease-prone regions of Karnataka, with 34–59% yield loss translating into a production loss of 1.05 lakh metric tonnes (MT) of arecanut [28].

The occurrence of FRD is increasing year after year due to the build-up of inoculum, shifts in the cropping system, and area expansion towards non-traditional tracts of Karnataka, resulting in extreme economic losses. In the endemic areas of India, FRD occurs immediately after the southwest monsoon showers and prevails until mid-September due to congenial environmental conditions [22]. FRD seems to exist in the predominant arecanut growing areas, which receive heavy rainfall during the southwest monsoon season [29]. FRD of arecanut is widely distributed in Karnataka, Kerala, Tamil Nadu, parts of the Andaman Islands, and Meghalaya. Concurrent occurrence of the pathogen was reported in different endemic areas of Kerala and Karnataka [30,31,32].

The status of FRD in arecanut has been changing rapidly, and the frequent and severe occurrence of this disease in arecanut growing regions is a serious matter of concern for growers. Up-to-date spatial information is the prime factor required to develop and maintain monitoring approaches for FRD and is necessary for quantification of the risks associated with the disease. This information could further help in the optimization of management practices [33]; therefore, knowledge of the spatial distribution of the disease as mapped for risk-prone areas is considered as a basic prerequisite to understand the consequences of disease development and disease monitoring and to design proper management approaches.

In recent years, geostatistical analysis tools have been utilized in the field of pathology to estimate the threat that occurs due to diseases, with device sampling approaches being used to investigate the prevalence of diseases and to identify disease-prone areas for site-specific disease management. Understanding the spatial distribution of FRD can help in the determination of inter-relationships between inoculum build-up and disease severity, the influence of abiotic factors on pathogen dynamics, and in risk assessment of disease in plants other than arecanut [34,35,36,37,38]. Different spatial disease distribution analysis methods are used to characterize the spatial positions of pathogens and locations of disease-affected gardens [39,40,41,42,43]. Spatial autocorrelation is generally used to determine the correlation between spatial data at different distance intervals in order to derive spatial dependence [42,44].

Geostatistical methods help by detecting, estimating, and mapping the spatial patterns of disease variables by focusing on interpolation, modeling, and interpretation of semivariograms [45]. Some earlier reports estimated the spatial distribution by employing kriging and interpolation techniques to assess the explicit spatial distribution of diseases on plantation crops other than arecanut [42,46,47,48]. Kriging approach was utilized to map spatial patterns of dead trees, showing that kriging is an accurate tool that can be used to estimate the spatial distribution of diseases [49].

There is a lack of reports on geostatistical estimates of FRD severity in arecanut crops across the major growing tracts of Karnataka, India. The present investigation was planned with the following objectives: (i) to determine the current status and spatial distribution of FRD across arecanut growing areas in Karnataka; (ii) to delineate the FRD hotspots or clusters using point pattern analysis; (iii) to estimate the risk associated with FRD in Karnataka using the indicator kriging approach.

## 2. Materials and Methods

### 2.1. Study Area and FRD Sampling

**Figure 1:**
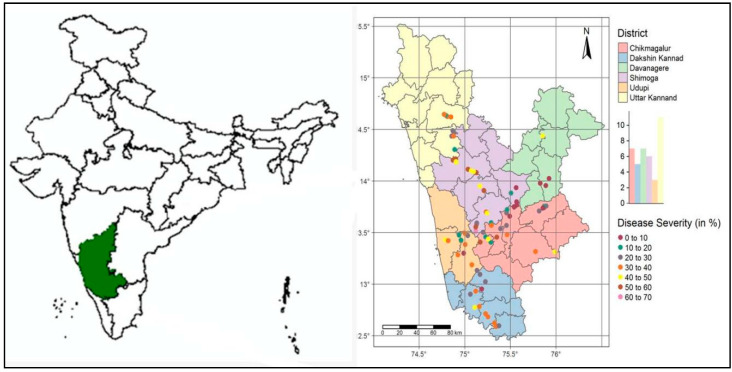
Featured map of India (left), Karnataka (green colored), and locations monitored for fruit rot disease (FRD) in Karnataka (right). Different colored circles (*n* = 83) represent FRD sampling sites in Karnataka during the monsoon seasons of 2018 and 2019.

This study was undertaken in the major arecanut growing areas of Karnataka (covering Malnad, coastal, and Maidan tracts), India, during the *Kharif* seasons (June to September) in the years 2018 and 2019 (Figure 1). About six districts were covered during the exploratory survey, consisting of predominant arecanut growing areas across 18 administrative taluks of Karnataka, India. Among the different districts studied, Shimoga, parts of North Canara (Uttar Kannad), and Chickmagalur districts belonged to the Western Ghats and hot–humid zones, with an average altitude of 1119 m and annual rainfall of 1812.5 mm, while South Canara (Dakshin Kannad) and Udupi districts fell under the coastal plain and hot–humid zones located at an altitude of 69 m and annual rainfall of 2599 mm. Transitional parts of Shimoga, Chickmagalur, and Davanagere districts were grouped in the sub-humid plain zones, with a mean altitude of 631 m and annual rainfall of less than 1000 mm.

FRD sampling was carried out across the arecanut growing districts of Karnataka from August to September, coinciding with the occurrence of disease under field conditions. A minimum distance of 5–10 km was maintained between each sampling site. From each administrative taluk, 5 to 10 samples were randomly drawn to maintain heterogeneity in the sampling procedure [27,50]. At each sampling site, 50 FRD-infected fallen nuts were randomly collected from three adjoining arecanut gardens. Each sampling site was geopositioned and infected arecanuts were labeled and stored in polythene covers. In total, 83 samples were collected across six districts belonging to major arecanut growing regions in Karnataka (Figure 1).

### 2.2. Determination of Disease Variables

During the investigation, 50 arecanut palms were randomly analyzed each year for the occurrence of FRD on tender nuts. Any symptoms suggestive of FRD, based on the literature review [20,22,51], were taken into consideration. The severity of FRD indicated by the proportions of infected palms among the total observed palms and the percent disease severity values of FRD was estimated using the following formula and a 1–6 rating scale, as described in Table 1 [52].

(1) $$\mathrm{Percent}\;\mathrm{disease}\;\mathrm{severity}\;\left(\mathrm{PDS}\right)=\frac{\mathrm{Sum}\;\mathrm{of}\;\mathrm{numerical}\;\mathrm{ratings}}{\mathrm{Total}\;\mathrm{number}\;\mathrm{of}\;\mathrm{plants}\;\mathrm{observed}\times \mathrm{Maximum}\;\mathrm{grade}}\times 100$$

### 2.3. Statistical Pre-Processing

The normal distribution of the underlying data is a prime assumption that is made before geostatistical analysis; therefore, we initially assessed the normality of the data by checking the Kolmogorov–Smirnov test [53], which was further confirmed using histograms and normal QQ plots to remove the slight global trend observed in the data. Percent severities of FRD sampled across different districts or taluks were subjected to the Kruskal–Wallis test in R statistical software (version R-4.0.3, R Core Team, 2020 [54]) to determine significant variations between administrative taluks in Karnataka state, India. Box and whisker plots representing percent severities of FRD sampled in different taluks were also constructed using R software (version R-4.0.3).

Agglomerative hierarchical cluster analysis using the average linkage method was performed based on percent severities of FRD to infer the distances among the taluks [55]. Optimization of the data and cluster analysis were accomplished through the ‘hclust’ function in R software (version R-4.0.3). In an average linkage hierarchical clustering, the distance (*L*) between two clusters (*r*, *s*) represents the distance between each point of a cluster to each point of the other cluster and can be expressed as follows:

(2) $$L\left(r,s\right)=\frac{1}{n_{r}n_{s}}\overset{n_{r}}{\underset{i=1}{\sum }}\overset{n_{s}}{\underset{j=1}{\sum }}D\left(X_{ri\;,}X_{sj}\right)$$

where *X* and *Y* are the observations from clusters *r* and *s*, respectively.

### 2.4. Geostatistical Analysis

The spatial distribution of the FRD occurrence across the studied districts of Karnataka was examined by employing two different geospatial statistical approaches, namely point pattern and surface interpolation analysis. The existing significant clusters of FRD in Karnataka were identified and confirmed using point-pattern-optimized hotspot analysis and Ripley’s K function. To generate spatial maps of the predicted surface and risk associated with FRD occurrence, inverse distance weighting (IDW), indicator kriging (IK), and ordinary kriging (OK) approaches were employed.

#### 2.4.1. Point Pattern Analysis

To determine point pattern analysis of the FRD across the studied areas of Karnataka, the degree of spatial dependency between neighboring plots consisting of infected palms was taken into account. Spatial autocorrelation analysis was carried out using local Moran’s *I* statistical analysis or local indicator of spatial association (LISA) statistics followed by optimized hotspot analysis [47,56,57]. In this analysis, the nearest neighbor sampling locations were taken into consideration [44,58]. The LISA for each observation gives an indication of the extent of significant spatial clustering of similar values around that observation [59]. The LISA, which is inferred using the *p*-value, indicates the presence of spatially significant clusters, while the following equation was used to compute Moran’s I statistic for a real unit *i*.

(3) $$\;I_{i}=Z_{i}\overset{n}{\underset{j}{\sum }}W_{ij}Z_{j}$$

where *I* is the statistic for district *i*, while *Z* is the difference between the FRD severity risk at *i* and the mean FRD severity for regions. *W* is the spatial weights matrix, which in this case only considers neighbors that share a common border or vertex. The *p*-values of $I_{i}$ that exceed the threshold indicate positive spatial autocorrelation, whereby identical values—either high or low values—are spatially clustered around point i. The *p*-values of $I_{i}$ below the threshold indicate a negative spatial autocorrelation, in that neighboring values are dissimilar or dispersed to the value at point i, while the remaining case depicts the presence of randomness; however, in particular sampling sites consisting of higher severity of FRD and for the nearest gardens possessing higher disease severity values, the particular location is considered as a hotspot or risk area [60].

Ripley’s *K*(*r*) function [61] was analyzed to confirm the significant clusters and spatial patterns of FRD, which is a widely used tool in ecological and epidemiological studies to examine distances at which spatial clustering or dispersion occurs [39,43,62]. Ripley’s K function was shown to be appropriate for analyzing point processes at different distances and was used to estimate the spatial pattern of FRD in Karnataka. Ripley’s *K(r)* is a tool used to analyze a mapped spatial point process; *K(r)* denotes the characteristics of point events over a range of scales. The assumption is that as the distance increases, each feature will typically have more neighbors. The function is expressed as *K(r) = λ − 1E*, where *E(r)* is the expected mean number of points within a distance *r* of randomly chosen points and *λ* is the FRD severity of the studied gardens.

#### 2.4.2. Spatial Surface Interpolation

The spatial interpolation approach is used to predict the known values of spatial information at other unknown sampling points. The percent severities of FRD at sites (*X*_1_, *X*_2_,…, *X_n_*) are (*Z*_1_, *Z*_2_,…, *Z_n_*); the purpose of spatial interpolation is to estimate the *Z* values at new points of *X*. Inverse distance weighting (IDW) and ordinary kriging (OK) are important basic techniques used to determine the estimated surface across the area of study.

The IDW method is a deterministic interpolation approach, whereby weighted means of the closest points are taken into account and weights are inversely proportional to the power of the distance [63,64]; therefore, the measured observations that are spatially nearest to the prediction location will have a higher influence on the predicted value. The IDW assumes that each measured observation has a local influence that diminishes with distance [65]. The IDW at an unsampled site *i* can be expressed as follows:

(4) $$\;F\left(i\right)=\overset{m}{\underset{i=1}{\sum }}W_{i}Z\left(r_{i}\right)={\frac{\sum_{i=1}^{m}Z\left(r_{i}\right)/\;\left|r-r_{i}\right|}{\sum_{j=1}^{m}1\;/\;{\left|r-r_{j}\right|}^{p}}}^{p}$$

where *P* is a parameter and *m* indicates a number of neighboring points taken into account at a certain cut-off distance. The interpolated values are then compared with the actual values from the omitted point via leave-one-out cross-validation. The performance of the interpolator is summarized by computing the root mean square residuals from the error (RMSE).

From a theoretical point of view, kriging is an interpolation technique for unbiased estimates of variables at unsampled spatial locations [66]. In kriging, the experimental variogram is utilized to estimate the spatial correlation of the random function *Z*(*X*_0_), while the spatial prediction of the values of variable *Z* at an unsampled point *X*_0_ is achieved using the following formula [67].

(5) $$\gamma \left(d\right)=\frac{1}{2}\sum {\left\{\left[Ẑ\left(X_{1}\right)-Z\left(X_{2}\right)\right]\right\}}^{2}$$

The surface maps of the severity of FRD were constructed using the ordinary kriging (OK) technique, which can be mathematically expressed as follows:

(6) $$Ẑ\left(X_{0}\right)=\overset{n}{\underset{i=1}{\sum }}\lambda_{i}Z\left(X_{i}\right)$$

where *Z* is the variable of interest at spatial coordinates *X_i_* and *X*_0_; *n* indicates the number of neighbors associated with the sampling point; *λ_i_* is the weight associated with sampling point *X_i_* and the *i*th observation point [68,69]. Before performing kriging, the percent severity of the FRD dataset distribution was confirmed using histograms and normal QQ plots. After fitting the semivariograms with different models, the semivariogram fitted with the exponential model was obtained as the best one. Using this semivariogram model information, OK-kriged maps were generated.

The semivariogram calculates the closest neighbor index based on the average spatial variability [70,71] and the FRD percent severity for each feature. A semivariogram is a function that describes the degree of spatial dependence within the data, which is defined as:

(7) $$ŷ\left(h\right)=\frac{1}{2N\left(h\right)}\overset{N\left(h\right)}{\underset{i=1}{\sum }}{\left[Z\left(X_{i}\right)-Z\left(X_{i}+h\right)\right]}^{2}$$

where *γ*(*h*) is the semivariance for the interval distance class *h*, *N*(*h*) is the number of data pairs of a given lag interval distance and direction, *Z* (*x_i_*) is the measured sample value at point *i*, and *Z* (*x_i_* + *h*) is the measured sample value at position *I* + *h*. Semivariogram values are fitted with various experimental models, such as exponential, spherical, and Gaussian models; the mathematical expression of these models is as follows.

Spherical model:

(8) $$ŷ\left(h\right)=C_{0\;}+C\left[1.5\frac{h}{a}-{\left(\frac{h}{a}\right)}^{3}\right],\;if\;0\leq h\leq a.\;$$

Exponential model:

(9) $$ŷ\left(h\right)=C_{0\;}+C\left[1-\exp\left\{-\frac{h}{a}\right\}\right]for\;h\geq 0\;$$

Gaussian model:

(10) $$ŷ\left(h\right)=C_{0\;}+C\left[1-\exp\left\{-\frac{h^{2}}{a^{2}}\right\}\right]for\;h\geq 0\;$$

*C*_0_, (*C* + *C*_0_), and *a* in the above semivariogram models are the nugget, sill, and range, respectively; for exponential and Gaussian models, *a* represents the theoretical range. To assess the accuracy of the estimates across the applied models and methods, the observed and estimated data were critically compared by deriving a set of accuracy measures, including the mean square error (MSE), root mean square error (RMSE), and average standard error (ASE). Finally, the percent severity of the kriged maps of FRD was constructed and symbolized accordingly.

An indicator kriging (IK) tool was used to unravel the disease-prone or uncertain areas where the disease severity of FRD on arecanut was more than 20 percent per garden [72,73,74,75]. An FRD severity level of more than 20% was considered to generate the probability risk maps based on the yield loss and economic threat that occurred due to disease. Indicator kriging was performed in the same manner as for ordinary kriging, as explained above, then a color-coded kriged map was generated, with contour symbolization representing higher risk areas of FRD in Karnataka.

## 3. Results

### 3.1. The Extent of FRD on Arecanut Samples across the Studied Areas of Karnataka

**Figure 2:**
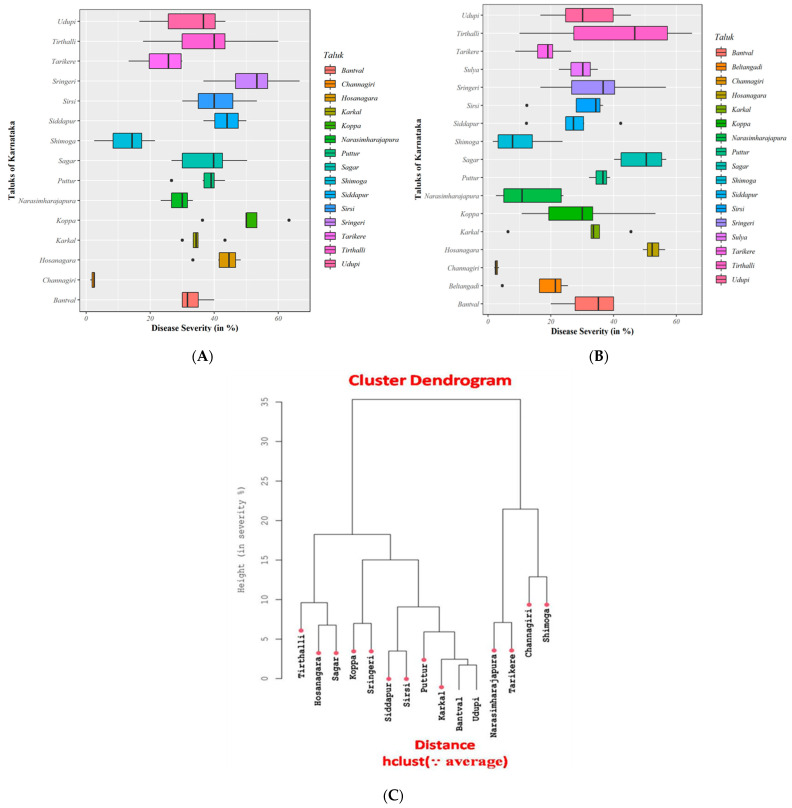
(**A**) Box and whisker plots indicating percent severity rates of FRD on arecanut samples across taluks in Karnataka during 2018. Middle bar = median; box = interquartile range (25th–75th percentile); whiskers (error bars) above and below the box = 90th and 10th percentile, respectively; dots = outliers. (**B**) Box and whisker plots indicating percent severity rates of FRD on arecanut samples across the studied taluks in Karnataka during 2019. Middle bar = median; box = interquartile range (25th–75th percentile); whiskers (error bars) above and below the box = 90th and 10th percentile, respectively; dots = outliers. (**C**) Agglomerative hierarchical cluster analysis of FRD using the average linkage method identified four main clusters among 18 taluks in Karnataka, India.

The pathogen was found throughout the evaluated areas of Karnataka, with samples exhibiting significant differences across the various districts studied during the monsoon seasons of 2018 and 2019. In 2018, the highest FRD percent severity was recorded in the Sringeri taluk of the Chickmagalur district, followed by Tirthalli, Sirsi, and Koppa taluks belonging to the Shimoga, North Canara (Uttar Kannad), and Chickmagalur districts, respectively (Figure 2A). The Lowest FRD occurrence was noted in Channagiri and Shimoga taluks of Davanagere and Shimoga districts of Karnataka (Figure 2A).

The FRD percent severity during 2019 revealed considerable variation among the evaluated taluks in Karnataka, with the highest FRD severity observed in Tirthalli taluk in Shimoga district, followed by Sagara and Sringeri taluks in Shimoga and Chickmagalur districts of Karnataka. Among the studied taluks, Channagiri in the Davanagere district presented the lowest severity of FRD (Figure 2B).

The agglomerative hierarchical cluster analysis of the severity of FRD among the 18 evaluated taluks belonging to six districts of Karnataka identified four main clusters using the average linkage method (Figure 2C). Tirthalli, Hosanagara, and Sagara formed a cluster, while the second cluster comprised Sringeri and Koppa taluks. The second cluster consisted of two sub-groups or clusters, which included Siddapura, Sirsi (comprising the 1st sub-group), Puttur, Karkal, Bantval, and Udupi (comprising the 2nd sub-group). The third and fourth clusters consisted of four taluks, with a clear separation between Channagiri, Shimoga, and other members of the cluster due to lower severity rates of FRD (Figure 2C).

### 3.2. Spatial Point Pattern Analysis of FRD in Karnataka

**Figure 3:**
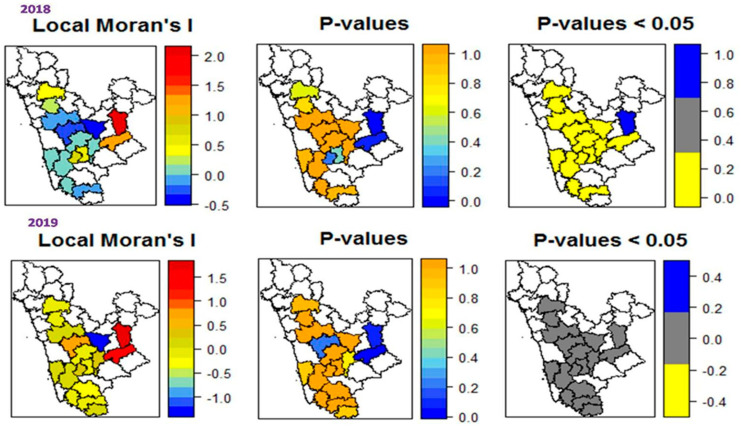
Local Moran’s I clusters across the studied areas in Karnataka, as inferred from *p*-values (*p* < 0.05), portraying higher, medium, and lower spatial rates of dependency.

The local Moran’s I spatial autocorrelation (LISA) cluster analyses identified different patterns of FRD at the district and taluk level during each of the two periods, representing random, dispersed, and aggregated clusters of severity surrounded by other areas (Figure 3). During 2018, higher spatially dependent clusters were identified in North Canara (Uttar Kannad), Shimoga, and parts of Chickmagalur districts, while the remaining districts presented lower spatial autocorrelation clusters. Considering the *p*-values (*p* < 0.05), all of the studied districts showed moderate spatial dependence (*p* < 0.05), indicating statistically insignificant clusters with randomness across most areas, although parts of Chickmagalur contained significant clusters (*p* = 0.2). During 2019, most of the areas show low clustering in the analyses, illustrating an absence or low levels of FRD severity among districts and taluks across Karnataka. A similar spatial pattern was observed in transitional (Maidan) tracts of Karnataka consisting of moderate clusters (*p* = 1), which showed relatively significant severity of FRD compared to neighboring districts. LISA analyses revealed that traditional arecanut growing areas presented a dispersed pattern of FRD on arecanut plantations, while the non-traditional tracts presented a significantly clustered pattern with an increased amount of FRD, indicating the potential spread of the disease to non-traditional areas.

**Figure 4:**
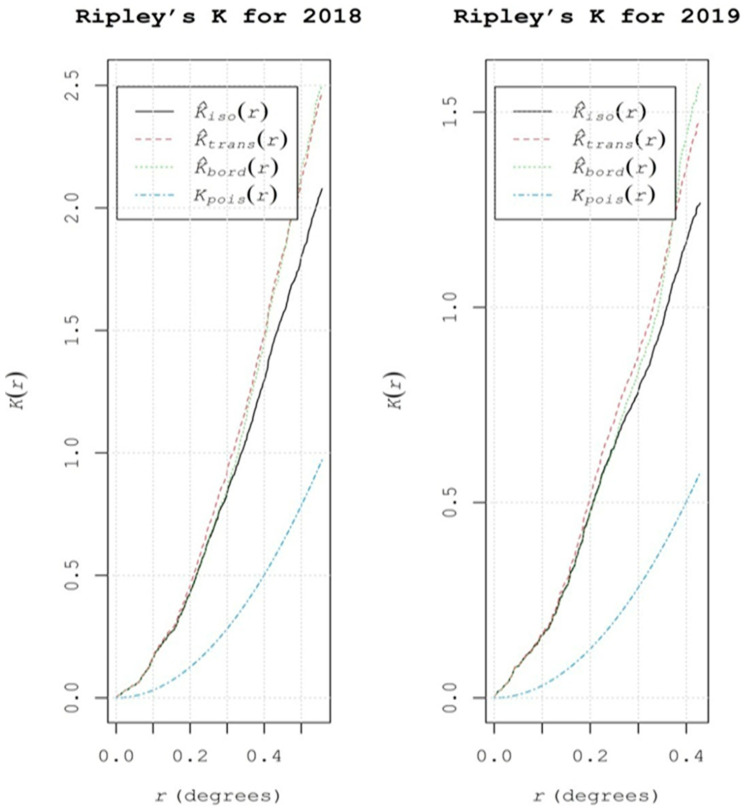
Ripley’s K function values for different sampling sites, showing the explicit spatial patterns of FRD in Karnataka during 2018 and 2019.

To demonstrate the exact spatial point pattern of FRD, we further analyzed Ripley’s K function, which characterized the point patterns by computing the average numbers of neighboring features associated with each feature at specific distances. Ripley’s K value allowed us to graphically examine the distances (in degrees) at which clusters occur. Both years of the study showed similar trends in terms of spatial patterns (Figure 4). The red line in Ripley’s K function plots represents translation correction, the blue line represents theoretical Poisson fitted data (expected), while the black line represents the observed data and shows the degree of point process clustering for different distance classes. Regarding the appearance of infection in 2018 and 2019, all distances (in degrees) displayed significant positive values, indicating that the observed disease patterns were moderately clustered. As the evaluation distances increased, each feature showed a greater number of neighbors. The average numbers of neighbors at distances of 0.2 and 0.4 were greater than the average concentrations of features throughout the study area, representing a moderate clustered distribution. Generally, moderately aggregated patterns of FRD were observed, although the distribution of infected sampling clusters varied slightly between years. In other words, there were no clusters of diseased sampling sites that consistently occurred in similar places over time or across seasons. The point pattern analysis of FRD via LISA and Ripley’s K function suggested the presence of significant hotspots in the Western Ghats and coastal parts (seashore), including non-traditional areas of FRD in Karnataka.

### 3.3. Surface Interpolation Approaches Used to Unravel the Spatial Distribution of FRD in Karnataka

#### 3.3.1. IDW Surface Interpolation

**Figure 5:**
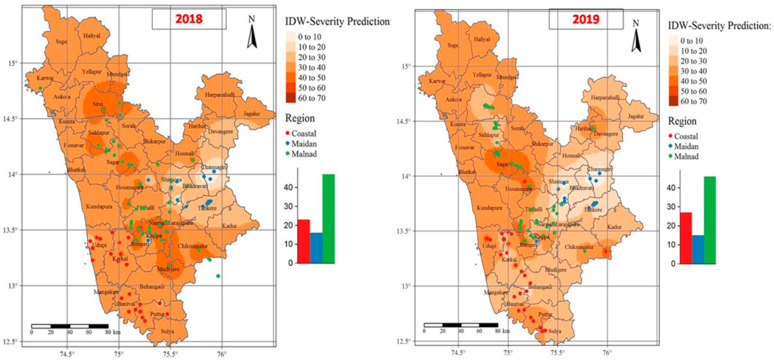
The optimized interpolated disease severity maps of FRD were generated using the inverse distance weighted (IDW) deterministic tool. Darker to lighter colors indicate higher to lower disease severity.

The point data were represented by complete enumeration of discrete observations, i.e., the FRD percent severity rates that occurred at discrete locations within the study areas. In Figure 5, the sampled points and values are superimposed on top of an (IDW) interpolated raster generated with a power coefficient of 15. The outcome of the IDW interpolation was depicted through color-coded maps of datasets for both years, with darker colors portraying higher percent severity rates of FRD. The interpolated surface areas of FRD differed considerably, indicating that the occurrence of disease was not consistent over the two consecutive years. During 2018 and 2019, more than 50 percent of FRD was mapped in 10.25 and 11.30% of the total interpolated surface, respectively. Furthermore, 55.45 and 62.75% of the interpolated surface exhibited FRD severity rates ranging between 30 and 50% during 2018 and 2019, respectively. The remaining interpolated area (44.35%) exhibited up to 30% FRD severity during 2018, while 26.95% of surfaces showed up to 30% disease severity in Karnataka during 2019. The predicted surface areas where the FRD percent severity rates exceeded 50% were mostly in Malnad tracts (parts of Shimoga, Chickmagalur, and North Canara) located near the Western Ghats, which experienced heavy rainfall, higher altitudes, and high humidity, along with areas near the seashore (Udupi and South Canara (Dakshin Kannad)) in Karnataka. About 11.25% of the predicted surfaces showed serious rates of FRD.

**Figure 6:**
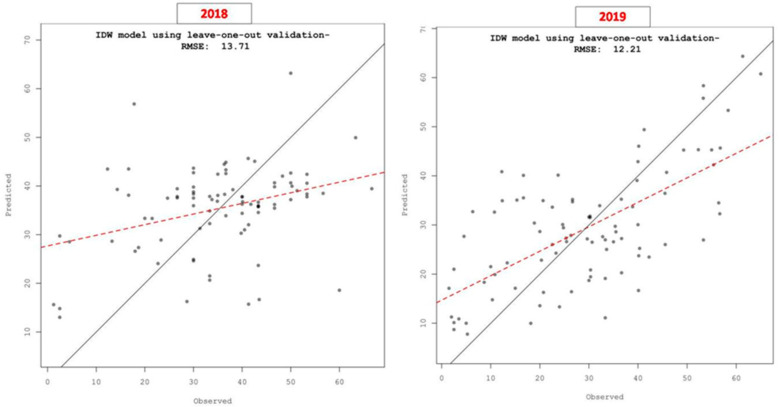
Scatter plot pitting predicted values vs. observed values at each sampled location for FRD in Karnataka following a leave-one-out cross-validation analysis.

To cross-validate the results of the IDW, we created a scatter plot of the predicted versus observed FRD percent severity values from our dataset (Figure 6). The solid diagonal line in the plot represents the one-to-one slope, while the red dashed line indicates the linear fit to the points, which helps to differentiate the pattern generated by the points. The leave-one-out cross-validation analysis revealed the differences between the observed (% severity of FRD at discrete locations) and predicted values using the IDW method. Figure 6 indicates that the predicted percent severity was biased in comparison to the observed data due to the mismatch of the solid line graph with the observed FRD percent severity values. Further, we explored the accuracy of the interpolators by computing the RMSE values of the model. The mapped results of the FRD during 2018 and 2019 across the study areas in Karnataka differed significantly, showing RMSE values of 13.71 and 12.21, respectively. Cross-validation results showed that the IDW interpolation tool used for estimation of patterns based on the FRD percent severity associated with arecanut plantations was more accurate.

#### 3.3.2. Semivariance Model and Ordinary Kriging (OK)

**Figure 7:**
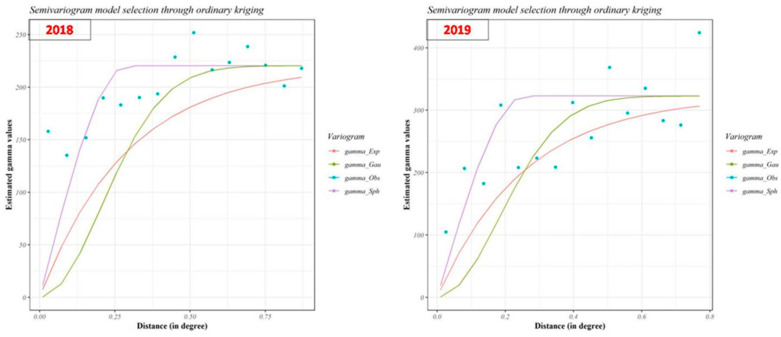
Semivariogram of different experimental models of FRD severity in 2018 and 2019. OK maps were generated using model information. The colored lines and blue dots represent the different models and model parameters.

Spatial patterns of percent FRD severity observations were determined by applying semivariogram experimental models, such as spherical, exponential, and Gaussian models, with other model parameters. The semivariogram cloud confirmed that the dataset was not affected by directional influences; hence, anisotropy was not taken into account while performing kriging. Among the different experimental models used for the 2018 data (Figure 7), the spherical model showed the best fit based on cross-validation of the semivariogram results (Table 2). The better fit of the spherical model was due to the cross-validation results exhibiting lower mean square error (MSE = 195.0087), root mean square standard error (RMSE = 13.9646), and average standard error (ASE = 0.3069) values. The nugget, range (in degrees), and partial sill values were found to be 0.5, 0.290479, and 220.4074, respectively, using a fitted model. For the data for 2019, the exponential model was found to be a better fit when considering results of the semivariogram cross-validation (Table 2). As was the case for the 2018 data, the better-fitting exponential model presented lower mean square error (MSE = 266.6116), root mean square standard error (RMSE = 16.3282), and average standard error (ASE = 0.4160) values. The nugget, range (in degrees), and partial sill values were found to be 0.5, 0.256481, and 322.8207, respectively.

**Figure 8:**
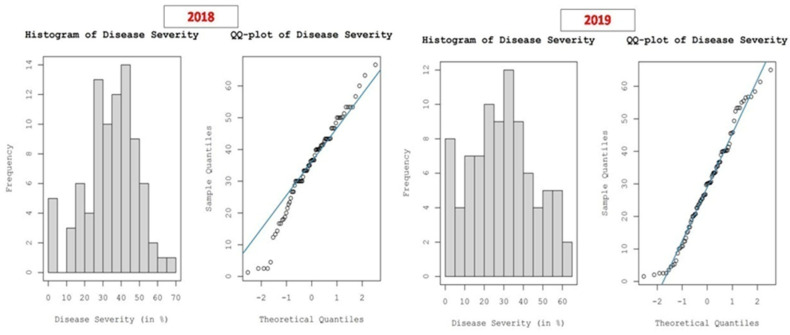
Histograms and normal QQ plots of FRD percent severity data used to understand the distribution of the dataset.

The FRD percent severity data collected across arecanut growing areas in Karnataka during 2018 and 2019 followed a normal distribution, as revealed by the Kolmogorov–Smirnov test. The normality of the dataset was further supported by histograms and normal QQ plots of the data (Figure 8). To remove the slight global trend in the data, the first-order nominal trend removal function was utilized prior to kriging and interpolation.

**Figure 9:**
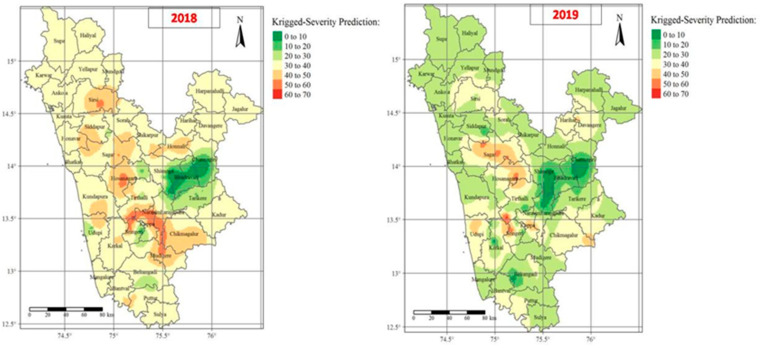
Interpolated maps representing distribution patterns of FRD in Karnataka during 2018 and 2019 using employing ordinary kriging (OK). Red to green color-coded surfaces depicts higher to lower FRD percent severity rates.

The spatial distributions based on the FRD percent severity across the investigated areas in Karnataka varied greatly from year to year and location to location during the 2018 and 2019. The highest FRD percent severity were observed in parts of the Malnad areas (traditional areas of Shimoga, Chickmagalur, and North Canara districts) as compared to non-traditional arecanut growing districts (parts of Davanagere, Shimoga, and Chickmagalur), which showed relatively lower proportions of FRD occurrence (Figure 9). As with the IDW interpolation technique, OK was used to explore spatial patterns of FRD on arecanut plantations by considering percent disease severity observations (*n* = 83). Ordinary kriged surface maps for 2018 and 2019 revealed that the maximum FRD severity rates were observed in certain parts of Shimoga, Chickmagalur, and North Canara, followed by arecanut plantations located near the seashore and in humid tracts (Udupi and South Canara). In the ordinary kriged maps for 2018 and 2019, about 8.30% and 5.65%, respectively, of the total interpolated surface area was predicted to show higher FRD severity, which even exceeded 50% disease prevalence in some areas.

#### 3.3.3. Semivariance Model and Indicator Kriging (IK)

Among the different experimental models evaluated, the spherical model fit the spatial distribution analysis of the FRD percent severity observations best by considering the model parameters and cross-validation results (Table 3). The spherical model returned lower mean square error (MSE = 208.1432), root mean square standard error (RMSE = 15.3264), and average standard error (ASE = 0.3865) Values. The nugget, range (in degrees), and partial sill values were found to be 0.5, 0.28417, and 228.9671, respectively, from the fitted spherical model (Table 3).

**Figure 10:**
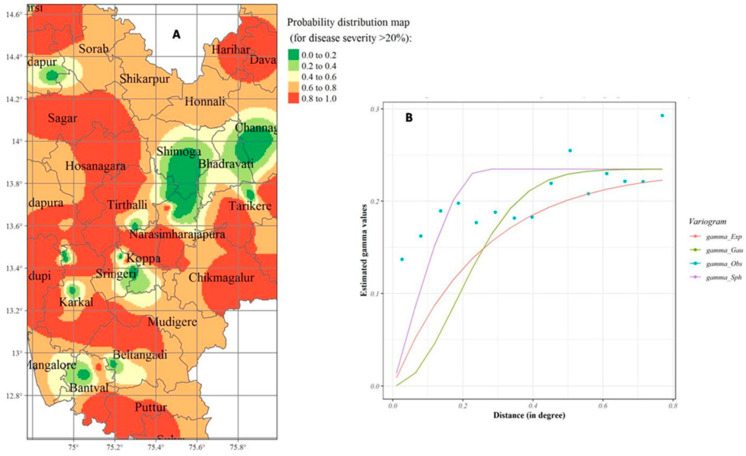
FRD probability distribution map for Karnataka (**A**) generated through semivariogram model information (**B**) using IK. The color-coded map (red to green) portrays the probability levels (high to low) of risk-prone areas infected with FRD.

Indicator kriging (IK) was analyzed similarly to ordinary kriging (OK). The probability distribution map (Figure 10) was prepared by considering the average FRD percent severity values during 2018 and 2019, along with the threshold value (FRD severity > 20%). The major high-risk areas included parts of traditional arecanut growing districts, namely Shimoga, Chickmagalur, Udupi, and South and North Canara. Even though the distribution of disease-prone areas changed temporally (2018 and 2019), the average disease severity values revealed that the higher uncertainty areas were mainly located in traditional arecanut growing regions, including (i) the high-altitude areas on the southern plateau, (ii) areas along the Western Ghats in hot–humid zone and in riverside locations, and (iii) the southern coastal tract of Karnataka along the seashore, which also has hot–humid conditions. Of the total interpolated surface area, around 50.5% of locations posed lower probability risk, which mainly included non-traditional arecanut growing tracts, such as the transitional areas of Shimoga, Chickmagalur, and Davanagere districts. Up to 35.6% of the predicted surface presented a moderate risk of FRD in Karnataka, while the remaining 13.9% of the interpolated area posed a very high probability (>50% FRD), which exceeded the threshold boundary (Figure 10).

## 4. Discussion

Arecanut is the predominant crop in southern Karnataka, particularly in the Western Ghats, coastal hot–humid plain, and transitional sub-humid zone. Since the first report on FRD in arecanut [22], the disease has occurred frequently in an endemic manner in traditional arecanut growing areas, including the Malnad tracts of Shimoga, Chickmagalur, North Canara (Uttar Kannad), Udupi, and South Canara (Dakshin Kannad). Over the years, FRD has been also occurred in non-traditional tracts in Karnataka in considerable proportions, posing serious threats to growers and indicating that FRD has started spreading to neighboring arecanut cultivation districts. The current status, explicit spatial distribution, and risk associated with FRD in Karnataka were examined using various geostatistical approaches. Spatial interpolation, spatial autocorrelation, and variogram analyses have been employed to understand the spatiotemporal distribution of cocoa pod rot disease in different countries [42,47,48]; however, spatial interpolation tools such as IDW, OK, and IK might be useful in understanding the spatial distribution of FRD across different districts in Karnataka.

This study represents the first attempt to map and demonstrate the local (district level) distribution of FRD occurrence in arecanut plantations across major growing locations in Karnataka, India. We employed global information system (GIS) tools in conjunction with spatial pattern and risk estimation methods to analyze the patterns of discrete percent FRD severity observations covering six districts and 18 administrative taluks in Karnataka over two consecutive years (2018 and 2019). The findings of the current study show to some extent the moderately similar I statistic values forming the spatial clusters in space and time. Additionally, our analysis results confirm that FRD was spatially distributed throughout most of the traditional areas in Karnataka, particularly the areas along the Western Ghats and seashore. These present study results supports the notion that FRD is present in serious proportions. This disease has inflicted huge economic losses to growers since the first report on FRD in 1910. The spatial point pattern analysis allowed us to identify FRD hotspots across arecanut growing districts in Karnataka. In addition, the surface prediction analysis using IDW, OK, and IK of FRD percent severity data revealed the higher probability (>50%) of FRD across Karnataka. The information generated from this study has a wider scope and implications in the development of management strategies and monitoring schemes for FRD.

Being an air- and soil-borne pathogen, the existence of moderate spatial clusters of FRD-infected gardens, as revealed by the local Moran’s I spatial autocorrelation (LISA), makes it possible that the dispersal of inocula (asexual spores which propagate over short distances through air currents) might have caused the spread of FRD to the neighboring gardens or adjoining areas from the initial infection [46,47]. Limiting their dispersal, therefore, is a top priority, particularly in non-traditional areas and younger gardens, in order to avoid severe epidemics. The results of this study show the moderate spatial distribution of FRD in arecanut crops across the study areas, which confirm the moderate spatial distributions in BPD observed for cocoa plantations across various study areas [42,48,76]. Infection hotspots in the Shimoga and Chickmagalur districts, including adjacent administrative taluks, could act as sources of future dissemination of FRD into non-traditional areas in Karnataka. Rapid dispersion of asexual spores and dormant distribution through soil could lead to increased build-up of inocula of the pathogen, as shown in these locations.

In many studies in the literature, point pattern and surface prediction analyses were employed to examine disease distribution patterns. Point pattern or hotspot analysis allowed us to recognize FRD hotspots among discrete observation points in Karnataka; in contrast, surface interpolation methods helped us to realize the predictable FRD percent severity rates in unsampled locations by generating smoothed surface maps. The existence of considerable FRD clusters or hotspots across different districts, including adjoining taluks in Karnataka, might help in the future in designing appropriate management approaches and in the proper monitoring of FRD. These results were consistent with previous studies on the use of point pattern geostatistical analysis to identify hotspots of rice sheath blight disease epidemics [77] and cocoa black pod rot disease [42,48].

During this study, we considered the percent severity of the FRD dataset generated through exploratory surveys to create the spatial distribution maps of FRD across investigated areas of Karnataka. As mentioned earlier, the FRD percent severity rates showed extensive temporally changes, resulting in deviations in the spatial distribution patterns as well; however, repeated surveys of FRD over many years might help us to establish the nature of FRD spatial patterns in Karnataka. The similar predicted surfaces of FRD across Karnataka were generated using deterministic surface interpolation tools, IDW, ordinary kriging (OK), and indicator kriging (IK). The experimental semivariogram of the FRD percent severity indicated relatively moderate spatial dependency. As the spatial clusters showed, kriging is a more suitable interpolation technique as compared to IDW, which has many drawbacks associated with distance-dependent interpolation; hence, the use of ordinary kriging (OK) is recommended to generate spatial distribution maps of FRD [78,79,80].

The semivariogram models revealed the distribution of FRD among the gardens evaluated across Karnataka and the presence of moderate spatial clusters and autocorrelation between the arecanut gardens, showing considerable prevalence rates of the disease, as well as how easily it spreads. Additionally, randomness was observed for several sampled gardens, which might be explained by the age differences of the palms. Over the years, the primary inoculum present in the garden would have had time to disseminate throughout the arecanut plantation, giving rise to the random pattern of diseased gardens. All of the experimental semivariogram models displayed moderate clustering of diseases gardens and indicated a large fraction of variance in the FRD percent severity rates. The results of this study are consistent with semivariogram models that displayed higher variance in cumulative rot rates of cocoa pod rot disease [77,81,82].

The limitation of ordinary kriging is that while generating smoothed maps, it does not consider extreme disease values [83]. To avoid this limitation, the indicator kriging technique was utilized to create probability distribution maps of FRD risk areas in Karnataka. The results from this study were in agreement with the findings for root-knot nematode infestation in cotton, whereby the risk maps were generated by considering the population densities through indicator kriging [84]. Similarly, modeling of the probability maps by observing disease uncertainty areas was achieved using the IK tool for different crops [72,73,74,75]. The probability risk maps generated in the present study show possible areas of uncertainty with maximum probability rates where the FRD severity has crossed the threshold level (severity > 20%). Due to a lack of information and ambiguity concerning the threshold level of FRD infection, we considered disease severity (>20%) as the benchmark for the generation of risk probability maps. The probability distribution maps created in this investigation could help farmers and the scientific community to identify areas of uncertainty due to FRD in arecanut plantations across Karnataka and to show where intervention tactics should be initiated.

## 5. Conclusions

The spatially explicit risk estimation and probability distribution maps generated in the current study will help in the development of management strategies for the disease in Karnataka. Additionally, this information could be useful for farmers and extension officers in setting up control measures and preventing further invasion of FRD into neighboring districts, regions, and states in the future. FRD in arecanut can be effectively managed by following preventative and curative approaches. In areas at higher risk of FRD infection, curative measures such as bunch spraying with Bordeaux mixture (1%) or any other oomycete-specific fungicide should be adopted to reduce inoculum build-up and further intraplot and intergarden spread. The paucity of knowledge among farmers regarding this century-old disease is a critical factor in its widespread distribution to non-traditional areas. Even though most of the farmers are aware of this century-old disease, there is a need to conduct training programs to increase awareness among the growers, especially regarding the manner in which that disease spreads, which will aid in the development of appropriate management strategies.

## Figures and Tables

**Table 1:** The following rating scale was used to estimate the percent disease severity of FRD on arecanut samples.

Rating Scale	Description
1	1–10% fallen nuts per palm
2	11–25% fallen nuts per palm
3	26–50% fallen nuts per palm
4	51–75% fallen nuts per palm + spread of the disease to bunch stalk
5	76–100% fallen nuts per palm + spread of the disease to the main stalk of the bunch
6	Complete crown death (CCD)

**Table 2:** Semivariogram experimental model parameters and cross-validation results. OK maps were generated using model information.

**2018**						
**Model**	**Range** **(in Degree)**	**Partial Sill** **(C + C_0_)**	**Nugget (C_0_)**	**MSE**	**RMSE**	**ASE**
Spherical	0.290479	220.4074	0.5	195.0087	13.9646	0.3069
Exponential	0.290479	220.4074	0.5	194.8522	13.9589	0.3102
Gaussian	0.290479	220.4074	0.5	196.9845	14.0610	0.4296
**2019**						
**Model**	**Range** **(in Degree)**	**Partial Sill** **(C + C_0_)**	**Nugget (C_0_)**	**MSE**	**RMSE**	**ASE**
Spherical	0.256481	322.8207	0.5	258.6190	16.0816	0.4401
Exponential	0.256481	322.8207	0.5	266.6116	16.3282	0.4160
Gaussian	0.256481	322.8207	0.5	271.4261	17.0589	0.5185

Legend: MSE: mean square error; RMSE: root mean square standard error; ASE: average standard error.

**Table 3:** Semivariogram experimental model parameters and cross-validation results. IK results were generated using model information.

Model	Range (in Degree)	Partial Sill (C + C_0_)	Nugget (C_0_)	MSE	RMSE	ASE
Spherical	0.28417	228.9671	0.5	208.1432	15.3264	0.3865
Exponential	0.28417	228.9671	0.5	209.0671	15.6622	0.3989
Gaussian	0.28417	228.9671	0.5	209.6384	15.8254	0.4066

Note: MSE: mean square error; RMSE: root mean square error; ASE: average standard error.

## Data Availability

The data presented in this study are available on request from the corresponding author.
